# ﻿Three new species and one new record of *Wilkinsonellus* Mason (Hymenoptera, Braconidae) from the Australo-Oriental region

**DOI:** 10.3897/zookeys.1240.153121

**Published:** 2025-06-05

**Authors:** Huan Li, Yuhao Meng, Geng Lu, Zhen Liu, Andrew Polaszek

**Affiliations:** 1 Changde Key Innovation Team for Wetland Biology and Environmental Ecology, Hunan University of Arts and Science, Changde 415000, China; 2 Science: Research, Natural History Museum, London, UK; 3 Zoology Key Laboratory of Hunan Higher Education, Hunan University of Arts and Science, Chande 415000, China

**Keywords:** Borneo, key, Microgastrinae, Solomon Islands

## Abstract

The small microgastrine braconid genus *Wilkinsonellus* Mason was known previously from parts of the Australo-Oriental region, but it has never been reported from Borneo (Brunei and Sarawak), Peninsular Malaysia, Seram, or the Solomon Islands before this study. Here we describe three new species, namely, *Wilkinsonelluscarinatus* Liu & Polaszek, **sp. nov.** from Brunei, Sarawak, and Seram; *Wilkinsonellusparacorpustriacolor* Liu & Polaszek, **sp. nov.** from Sarawak; and *Wilkinsonellusrugiscutum* Liu & Polaszek, **sp. nov.** from Peninsular Malaysia. We also present a new distribution record of *Wilkinsonellusamplus* Austin & Dangerfield from the Solomon Islands, along with a key to 21 species known from the Australo-Oriental region.

## ﻿Introduction

*Wilkinsonellus* Mason is a relatively small genus of the hyperdiverse microgastrinaes (Hymenoptera, Braconidae) with 23 described species, from all tropical areas of the world (Fernández-Triana et al. 2020). Species of *Wilkinsonellus* were placed in the *henicopus* and *daira*-group of *Apantelessensu lato* (Nixon 1965) prior to being elevated as a genus in Cotesiini by Mason (1981). Austin and Dangerfield (1992) reported three Australian species later and other papers published on this genus in the 21^st^ century include the following: Long and van Achterberg (2003, 2011), Vietnam; Zeng et al. (2011), China; Rousse and Gupta (2013), Réunion Island; Arias-Penna et al. (2013, 2014), Neotropics and Fiji; and Fernández-Triana and van Achterberg (2017), Arabian Peninsula. Several keys have been produced for regional or world species since it had been established (Long and van Achterberg 2003, 2011; Zeng 2012; Arias-Penna et al. (2013, 2014). The latest key (Arias-Penna et al. 2014) to 22 species from the world incorporated 18 known species from the Australo-Oriental region. However, prior to this study, no species of this genus have ever been reported from Borneo, Peninsular Malaysia, Seram, or the Solomon Islands.

This group was recently placed in Fernández-Triana et al.’s (2020)*Cotesia* group with 28 other genera, an informal grouping of Microgastrinae genera due to the fact that the tribes (sensu Mason 1981) did not properly reflect the phylogenetic relationships within this subfamily. It is very recognizable among these genera for the very long, narrow T1, the distinctive sculpture and carination pattern on the propodeum, and the strongly angled vein r with 2-SR in the fore wing (Mason 1981; Long and van Achterberg 2011; Arias-Penna et al. 2013, 2014). *Wilkinsonellus* is probably most closely related to *Diolcogaster* and *Parenion* (Austin and Dangerfield 1992).

There is one host record, that of *Microthyrisprolongalis* (Lepidoptera, Crambidae) for the Neotropical species *W.alexsmithi* (Arias-Penna et al. 2013). However, as members of Microgastrinae whose hosts cover almost the full range of families of Lepidoptera, *Wilkinsonellus* species have potential significance for biological control.

Here, as a part of our ongoing research on worldwide Microgastrinae, we describe three new species and provide a new distribution record based on specimens from the Natural History Museum, UK.

## ﻿Materials and methods

Descriptions and measurements were conducted using a Zeiss Stemi SV6 stereomicroscope. Photographs were captured with a digital camera (Zeiss AxioZoom or Hirox HRX-01) and processed using Helicon Focus software. Further image enhancements were done in Adobe Photoshop CS6. Morphological terminology for body structures and measurements primarily follows Austin and Dangerfield (1992), Long and van Achterberg (2011), and Arias-Penna et al. (2014). Wing vein terminology follows the modified Comstock–Needham system (van Achterberg 1993), while cuticular sculpture terminology follows Harris (1979). The following abbreviations are used in this study: **POL** = postocellar line; **OOL** = ocular-ocellar line; **OD** = ocellar diameter; **T1** = first metasomal tergite; T2 = second metasomal tergite; **T3** = third metasomal tergite. The new species described in this study are deposited in the Natural History Museum, UK (**NHMUK**). The holotype of *Wilkinsonellusamplus* is housed in the Australian National Insect Collection (**ANIC**), Canberra, Australia.

## ﻿Taxonomy

### ﻿Key to species of the genus *Wilkinsonellus* Mason from the Australo-Oriental region

Modified from Arias-Penna et al. 2014 and Long and van Achterberg (2011).

**Table:** 

1	Mesosoma flattened, scutellum at same level as propodeum (Fig. 1a); scutellum almost smooth and without a transverse posterior carina (Fig. 1e); metacoxa more or less shortened, not surpassing apex of T1 (Fig. 1f)	**2**
–	Mesosoma normal, scutellum protruding distinctly above level of propodeum (Fig. 3f); scutellum rugose or punctate-rugose or finely punctate (Fig. 3e), often with an apical spine (Fig. 4f); metacoxa long, distinctly surpassing apex of TI (Fig. 4k)	**3**
2	Propodeum with a very coarse median carina combined with various strong secondary rugae (Fig. 1e); body large (about 5.2 mm), completely brown-yellow (Fig. 1a) [Papua New Guinea]	***W.daira* (Nixon)**
–	Propodeum with a coarse median carina dividing propodeum into two smooth lateral parts, without rugae; body rather small (2.4 mm), black, metasoma yellow-brown, T1 ivory colored laterally [China]	***W.flavicrus* Long & van Achterberg**
3	Ocelli small or medium-sized, OOL more than diameter of posterior ocellus or subequal (Figs 3b, 4b), inner margins of eyes at antennal sockets hardly or not emarginate (Figs 3c, 4c)	**4**
–	Ocelli large to very large (Figs 2d, 5b), OOL less than diameter of posterior ocellus or equal; inner margins of eyes at antennal sockets deeply emarginate (Figs 2c, 5e)	**17**
4	Mesopleuron obliquely striate above precoxal sulcus; OOL 1.0–1.5× OD [Australia, Papua New Guinea]	***W.striatus* Austin & Dangerfield**
–	Mesopleuron smooth or sparsely punctate above precoxal sulcus (Figs 3f, 4f); OOL 1.6–2.4× OD (Figs 3b, 4b)	**5**
5	Body completely brown-yellow	**6**
–	Body partly dark brown or black, at least propodeum and mesopleuron (Figs 3a, 4a)	**10**
6	Face coarsely reticulate-rugose	**7**
–	Face finely, densely punctate	**8**
7	Body entire fulvous, except metafemur and metatibia slightly darkened at extreme apex; notauli rugose-reticulate [Philippines, China]	***W.iphitus* (Nixon)**
–	Body yellow-orange except tergites III–IV (medially) and following tergites dark brown, and metatarsus infuscate; notauli coarsely punctate [La Réunion]	***W.narangahus* (Rousse & Gupta)**
8	Hind wing with vannal lobe of typical microgastrine dimensions [Fiji]	***W.nescalptura* Arias-Penna, Zhang & Whitfield**
–	Hind wing with vannal lobe reduced	**9**
9	Outer dorsal surface of metacoxa with distinct longitudinal carina, inner dorsal surface coarsely reticulate; hypopygium smooth and hairless [Papua New Guinea, Australia]	***W.tomi* Austin & Dangerfield**
–	Outer dorsal surface of metacoxa with coarse and heterogeneous aerolate-rugose sculpture throughout without carina, ventral surface with dense and fine punctate those two areas separated by a flat, smooth and shiny stripe; hypopygium setose [Fiji]	***W.fijiensis* Arias-Penna, Zhang & Whitfield**
10	Scutellum with a small spine apically (Fig. 4f)	**11**
–	Scutellum without spine apically (Fig. 3f)	**16**
11	Head yellow-orange or red-brown (Fig. 4b)	**12**
–	Head black or black-brown	**15**
12	Frons with two distinct parallel carinae between antennal sockets [Vietnam]	***W.nigrocentrus* Long & van Achterberg**
–	Frons with rippled sculpture between antennal sockets (Fig. 4b)	**13**
13	Head and mesosoma red-brown; surface of metacoxa reticulate with fine granulate background sculpture [India]	***W.granulatus* Ahmad, Pandey, Haider & Shuja-Uddin**
–	Head yellow-orange and mesosoma brown-black (Fig. 4a); outer dorsal surface of metacoxa with coarse aerolate-rugose sculpture, but finely sculptured in the remaining area (Fig. 4f)	**14**
14	Vein m-cu of fore wing nearly half length of 2-SR (Fig. 4j); mesoscutum with small punctures, intervals uneven with minute punctuation and larger than puncture’s diameter (Fig. 4e); propodeum without transverse carinae (Fig. 4h) [Malaysia (Sarawak)]	***W.paracorpustriacolor* Liu & Polaszek, sp. nov.**
–	Vein m-cu of fore wing about as long as 2-SR; mesoscutum with dense areolate-rugose punctures; propodeum with several transverse carinae attaching medio-longitudinal carina [Fiji]	***W.corpustriacolor* Arias-Penna, Zhang & Whitfield**
15	Vein 1-CU1 of fore wing 0.50× as long as vein 2-CU1; pterostigma distinctly shorter vein 1-R1 (23: 60); frons smooth; propodeum largely rugose; vein cu-a of hind wing more or less sinuate [Vietnam]	***W.nigratus* Long & van Achterberg**
–	Vein 1-CU1 of fore wing 0.85× as long as vein 2-CU1; pterostigma as long as vein 1-R1; frons rugose-punctate; propodeum sparsely rugose apically, smooth basally; vein cu-a of hind wing curved [Vietnam]	***W.masoni* Long & van Achterberg**
16	Head, mesoscutum and scutellum black (Fig. 3b, e); vertex and temple densely punctate (Fig. 3b); punctures on scutellum umbilicate and deep (Fig. 3e) [Brunei, Malaysia (Sarawak), Indonesia (Seram)]	***W.carinatus* Liu & Polaszek, sp. nov.**
–	Head brown-yellow, mesoscutum and scutellum red-brown; vertex and temple almost smooth; punctures on scutellum rugose and shallow [Philippines]	***W.thyone* (Nixon)**
17	Lateral lobes of mesoscutum and mesopleuron ventrally yellow or brown-yellow (Figs 2d, f, 5b, c); ocelli strongly protuberant, in frontal view completely above dorsal level of eyes	**18**
–	Lateral lobes of mesoscutum and mesosternum dark brown or blackish; ocelli less protuberant, in frontal view partly below dorsal level of eyes	**20**
18	Metacoxa yellow or orange without dark brown patch (Fig. 2e); metafemur yellow to orange-brown on apical half (Fig. 2a); fore wing with vein r shorter than 2-SR (Fig. 2e) [Australia, Solomon Island]	***W.amplus* Austin & Dangerfield**
–	Metacoxa yellow with apex light brown or with a dark brown ventral patch (Fig. 5c); metafemur dark brown at least on posterior half (Fig. 5c); fore wing with vein r 1.4–1.5× longer than 2-SR	**19**
19	Notauli absent; fore wing with vein 1-CU1 over half length of 2-CU1; POL nearly as long as OD [Vietnam]	***W.longicentrus* Long & van Achterberg**,
–	Notauli impressed with rugose punctures (Fig. 5b); fore wing with vein 1-CU1 0.3× 2-CU1 (Fig. 5d); POL less than half length of OD (Fig. 5b) [Malaysia (Selangor)]	***W.rugiscutum* Liu & Polaszek, sp. nov.**
20	Temple narrow, in lateral view its width near middle of eye 0.3–0.35× transverse diameter of eye; OOL of female 0.2–0.3× OD; vertex without transverse rugosities [Vietnam]	***W.paramplus* Long & van Achterberg**
–	Temple wider, in lateral view its width near middle of eye 0.4–0.5× transverse diameter of eye; OOL of female 0.5× OD; vertex with distinct transverse rugosities [Vietnam]	***W.tobiasi* Long**

#### ﻿ Wilkinsonellus daira

Taxon classificationAnimaliaHymenopteraBraconidae

(Nixon, 1965)

8C641ED4-A5A8-5030-A2AD-21985E54C5BA

Fig. 1

Apanteles daira Nixon, 1965: 198. Holotype in NHMUK (examined).
Wilkinsonellus daira Mason, 1981: 122; Long and van Achterberg 2003: 221; Long and van Achterberg 2011: 124; Arias-Penna et al. 2014: 30; Fernández-Triana et al. 2020: 995.

##### Material examined.

***Holotype*** • 1♂; Keravat, New Britain; 10.v.1952; bred from *Hibiscus* leaf folder (J.H.Barett); Com.Inst.Ent.Coll. No.; 3017, B.M.TYPE HYM 3c.1884.

##### Diagnosis.

**Figure 1.**
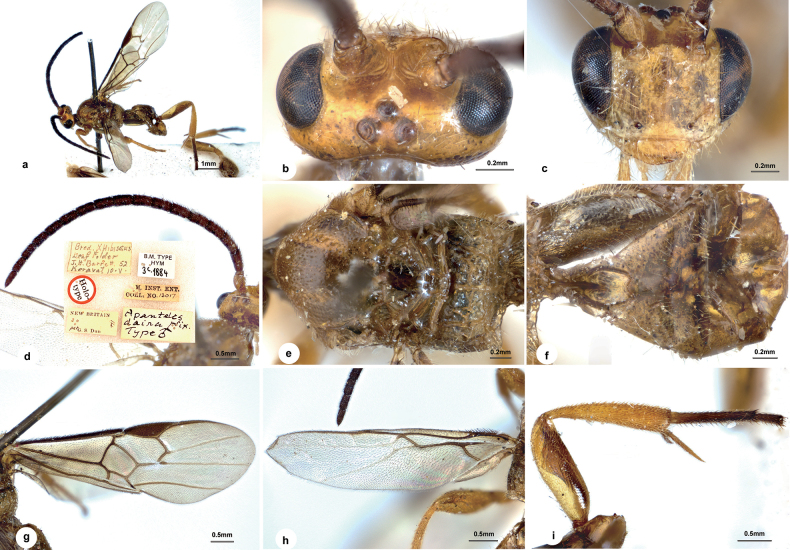
*Wilkinsonellusdaira* (Nixon, 1965), male, holotype **a** habitus, lateral view **b** head, dorsal view **c** head, frontal view **d** antenna and holotype labels **e** mesosoma, dorsal view **f** metasoma, dorsal view **g** fore wing **h** hind wing **i** hind leg.

**Male**: legs fulvous throughout except metatarsus infuscate (Fig. 1a, i); the transverse, posterior tangent to anterior ocellus not touching posterior pair; antennal sockets very deep, their anterior margins strongly raised, forming a sharp keel medially; head above strongly shiny with a few scattered punctures (Fig. 1b); face strongly transverse, very shiny with large, scattered punctures, with vague striation towards eye margin (Fig. 1c); mesoscutum very shiny with scattered punctures; notauli indicated by a row of larger, more irregularly shaped punctures; propodeum with strong medial carina and various, strong secondary carinae (Fig. 1e); vein 1-R1 about 1.5× longer than its distance from the apex of marginal cell (Fig. 1g); petiole of T1 linear but dilated posteriorly, with deep groove throughout; T2+T3 smooth, shiny; T2 with a hardly defined, raised median swelling that is elongate and more or less parallel-sided (Fig. 1f) (based on Nixon, 1965).

##### Notes.

Nixon (1965) mentioned that this species was characterized by its very large size, colour, and lobed claws. Based on our examination of the holotype, the body length is 5.2 mm, fore wing length is 5.3 mm (Fig. 1a), the body is entirely brown-yellow with head slightly paler (Fig. 1a, b). Other supplementary characters we add here to the holotype description: antenna distinctly shorter than body length, with penultimate flagellomere 2.1× as long as wide (Fig. 1d); fore wing with vein r 1.3× 2-SR, m-cu 0.9× 2-SR+M and 1.3× 2-SR, 1-CU1 0.6× 2-CU1 and 1.3× cu-a (Fig. 1g); hind wing with short setae on vannal lobe posterior margin, cu-a distinctly curved posteriorly (Fig. 1h).

#### ﻿ Wilkinsonellus amplus

Taxon classificationAnimaliaHymenopteraBraconidae

Austin & Dangerfield, 1992

34BCBD74-783F-53A3-B263-FC53373C3B70

Fig. 2

Wilkinsonellus amplus Austin & Dangerfield, 1992: 62. Holotype in ANIC (not examined but original description checked).
Wilkinsonellus amplus : Long and van Achterberg 2011: 125; Arias-Penna et al. 2014: 32; Fernández-Triana et al. 2020: 995.

##### Material examined.

• 1♀; Solomon Islands, Guadalcanal Island, Honiara; 13–16.ix.1953; J.D. Bradley, Rennell I. Expedition, B.M.1954–222, M[ercury].V[apour]; light; NHMUK101639756.

##### Diagnosis.

**Figure 2.**
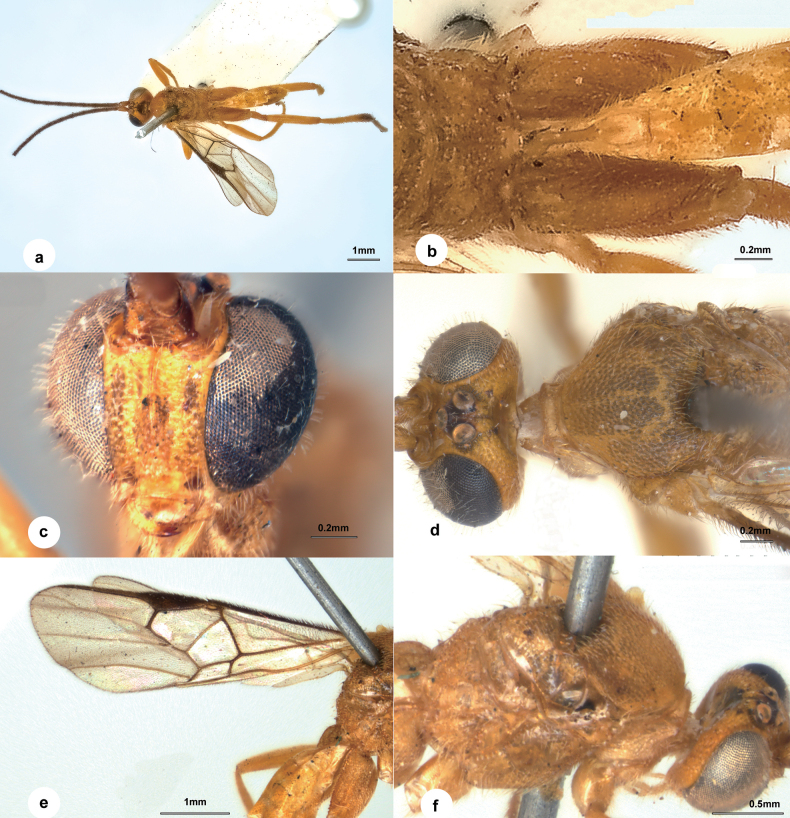
*Wilkinsonellusamplus* Austin & Dangerfield, 1992, female **a** habitus, dorsal view **b** propodeum and T1–T3, dorsal view **c** head, frontal view **d** head and mesosoma, dorsal view **e** fore wing **f** mesosoma, lateral view.

Similar to *W.tomus* except the following: body much larger, ♀ length 3.9–4.1 mm, ♂ length 3.5–3.9 mm; area between ocelli black (Fig. 2d); sometimes patch on posterior tergites and apex of metafemur, tibia and metatarsus dark brown to black; eyes very large, inner margins of eyes at antennal sockets deeply emarginate (Fig. 2c); face narrow, head across medial face 2.5× as wide as face (Fig. 2c); ocelli large, closer to each other than their own diameter, lateral ocelli raised above vertex on a low turret, separated from eye margin by 0.5× their own diameter (Fig. 2d); temple and occiput narrow; medial part of posterior band of scutellum rugose (based on Austin and Dangerfield 1992).

##### Distribution.

Australia, Solomon Islands (new record).

##### Note.

This species is easily distinguished from its congeners by its large size, large eyes, large and strongly protuberant ocelli, yellow mesosoma, deeply emarginated inner eye margins and more transverse head (Austin and Dangerfield 1992; Long and van Achterberg 2011). The specimen from the Solomon Islands is consistent with the descriptions and illustrations in the above references except for the larger body size (4.9 mm in length).

#### ﻿ Wilkinsonellus carinatus

Taxon classificationAnimaliaHymenopteraBraconidae

Liu & Polaszek sp. nov.

580E3C08-4965-5D98-A382-3C780B7BB3EC

https://zoobank.org/461F1A5D-20B5-499A-87E6-6095211E9942

Fig. 3

##### Diagnosis.

**Figure 3.**
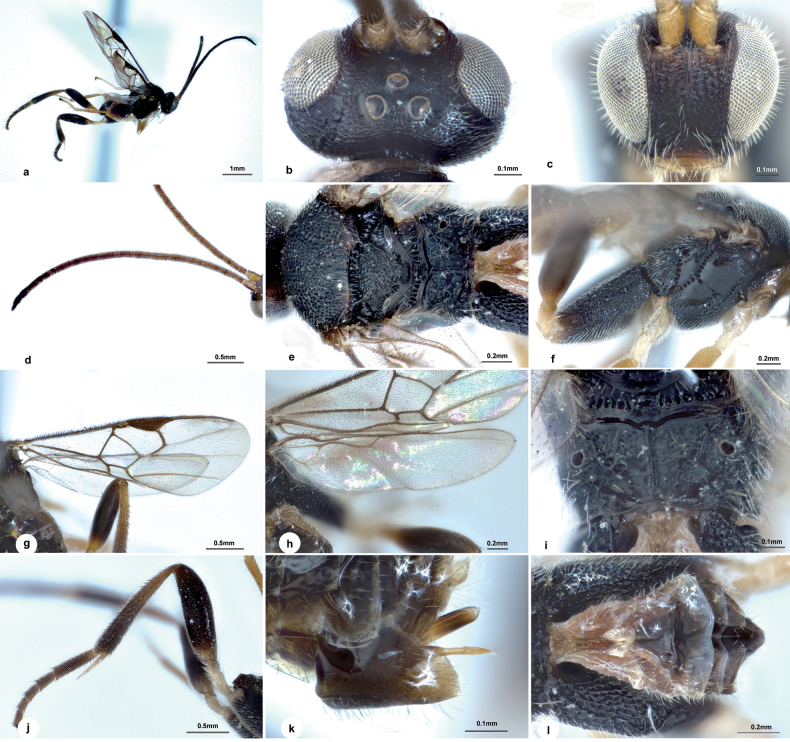
*Wilkinsonelluscarinatus* Liu & Polaszek, sp. nov., female **a** habitus, lateral view **b** head, dorsal view **c** head, frontal view **d** antenna **e** mesosoma, dorsal view **f** mesosoma, lateral view **g** fore wing **h** hind wing **i** propodeum, dorsal view **j** hind leg **k** ovipositor sheath **l** metasoma, dorsal view.

Body 2.7 mm long, black, except T1, lateral part of T2–T7 light brown (Fig. 3a); OOL: OD = 1.7 (Fig. 3b); face with minute punctures, their intervals uneven to transversely rugulose (Fig. 3c); antenna 1.3× as long as body length, with penultimate flagellomere 2.6× as long as wide (Fig. 3d); mesoscutum distinctly strigose with a medio-longitudinal carina posteriorly; scutellum with dense umbilicate punctation, protruding far above level of propodeum in lateral view (Fig. 3e); propodeum areolate-rugose along inner side of oblique carinae (Fig. 3i); mesopleuron largely smooth except densely punctate on anterior and upper edges (Fig. 3f); vein r 0.8× 2-SR(Fig. 3g); metacoxa extending nearly to apex of metasoma, with areolate-rugose sculpture on outer dorsal edge, nearly smooth medio-longitudinally, finely punctate ventrally (Fig. 3f); petiole of T1 smooth, 2.0× as long as basal width, 4.4× as long as medial width; T2 subtriangular, smooth, 1.8× as wide as length in the middle; T3 about as long as T2 (Fig. 3l); hypopygium broad (Fig. 3k).

##### Description.

**Female.** Body length 2.7 mm, fore wing length 3.2 mm (Fig. 3a).

***Head.*** 1.7× as wide as long, 0.8× as wide as mesoscutum. Eyes 1.7× longer than temple in dorsal view. Temple dull with small punctures, rounded behind eyes in dorsal view. Vertex dull with small sparse punctures, slightly transversely rugulose behind ocelli. Ocelli large, distance between fore and a posterior ocellus 0.6× as long as minor axis of a posterior ocellus, POL:OD:OOL = 1.0:1.0:1.7. Frons slightly depressed, with curved carinae around sockets (Fig. 3b). Face slightly shiny with minute punctures, their intervals uneven to transversely rugulose, slightly bulging medio-longitudinally, 0.9× as wide as high. Clypeus 2.3× as wide as medial length, nearly smooth with superficial punctures. Length of malar space 1.4× width of mandible (Fig. 3c). Antenna 1.3× as long as body length, with 1^st^, 2^nd^ and penultimate flagellomeres 3.3, 3.1, and 2.6× as long as wide, 1^st^ indistinctly longer than 2^nd^, flagellomeres gradually shortening towards penultimate flagellomere, closely articulated (Fig. 3d).

***Mesosoma.*** Length:width:height = 1.1:1.0:1.1. Mesoscutum dull with even punctures, which tend to be larger along the “imaginary” notauli, intervals uneven with minute punctuation and about the length of a puncture’s diameter, and distinctly strigose with a medio-longitudinal carina posteriorly. Scutellar sulcus wide and straight with carinae inside. Scutellum dull with dense umbilicate punctation, medial part of posterior band of scutellum rough, protruding far above level of propodeum in lateral view (Fig. 3e, f). Propodeum 2.8× wider than high, dull with a strong percurrent medio-longitudinal carina, and adjacent oblique carinae, which have weak transverse carinae between them, shallowly punctate anterio-laterally, areolate-rugose along inner side of oblique carinae; spiracles large, enclosed by costulae (Fig. 3i). Mesopleuron shiny, largely smooth except densely punctate on anterior and upper edges, sternaulus crenulate, with an oblique furrow attaching right-angled to its anterior end (Fig. 3f).

***Wings.*** Fore wing: pterostigma narrow, 3.4× as long as its widest part; vein 1-R1 1.4× length of pterostigma; vein r arising from apical third of pterostigma, slightly longer than maximum width of pterostigma, 0.8× 2-SR; vein m-cu 2.3× as long as 2-SR+M, nearly as long as 2-SR; vein 1-CU1 0.7× 2-CU1 and 1.7× cu-a (Fig. 3g). Hind wing: vannal lobe of normal size, posterior margin with short setae medially, basally and apically with longer setae, cu-a slightly curved (Fig. 3h).

***Legs.*** Metacoxa long, extending nearly apex of metasoma, areolate-rugose sculptures on outer dorsal edge, nearly smooth medio-longitudinally, finely punctate ventrally (Fig. 3f). Metafemur 3.4× as long as its widest part. Inner spur of metatibia ^4^/_5_ length of metabasitarsus. Metabasitarsus 0.7× as long as combined length of tarsomeres 2–5 (Fig. 3j).

***Metasoma.*** 0.9× length of mesosoma. Petiole of T1 smooth, bottle-shaped, attenuated to posterior edge, 2.0× longer than basal width, 4.4× longer than medial width, petiole with a groove reaching to half of apical swollen area. T2 subtriangular, smooth, 1.8× as wide as length in the middle. T3 about as long as T2, T3, and posterior tergites smooth and more delicate and membranous (Fig. 3l). Hypopygium broad, hardly reaching apex of metasoma. Ovipositor sheath slightly protruding beyond apex of metasoma, almost glabrous (except for a few extremely short hairs apically) (Fig. 3k).

***Colour.*** Body black, except T1, lateral part of T2–T7 light brown, dorsal part dark brown, and face slightly brown below sockets (Fig. 3a). Palpi white. Tibia spurs pale yellow. Antenna brown to dark brown except scape and pedicel yellow-brown. Fore and middle legs off-white to yellow-brown, hind leg black except trochanter pale yellow, extreme basal femur and basal third of tibia yellow-brown. Wing membranes hyaline, slightly infumate, fore wing with pterostigma brown, veins pale brown to brown.

**Variation.** Specimens from Brunei (Labi) with T1–T2 white laterally, and specimens from Brunei (Kuala Belalong), Sarawak and Seram with yellow-brown head. Body length 2.7–3.1 mm.

**Male.** Similar to female except T1 longer with petiole at least 5.5× longer than medial width and T2 narrower. Body length 2.7–3.1 mm.

##### Host.

Unknown.

##### Material examined.

(NHMUK) ***Holotype*** • 1♀; Brunei, Seria swamp forest edge; M.C. Day; 14.ii–4.iii.1982; NHMUK010639301. ***Paratype*** • 1♀; Brunei, Ulu Temburong N.P. (Malaise trap); M.C. Day; BMNH(E)2011-106; 14.ii–9.iii.1982; NHMUK010639751 • 2♀♀3♂♂; Brunei, Labi, mixed dipterocarp forest, 200m; viii–ix.1979; NHMUK010826306, 010826288, 010826315, 010826623, 010826324 • 4♀♀6♂♂; Brunei, Kuala Belalong FSC 4°34′N, 115°7′E; 1800m Malaise trap; iii.1992; NHMUK010826331, 010826319, 010826344, 010826346, 010826280, 010826347, 010826333, 010826329, 010826355, 010826349 • 6♀♀1♂; Malaysia, Sarawak (Borneo), 4^th^ Div. Gunung Mulu, RGS Exp.; 27.vi–19.vii.1978; H. Vallack; NHMUK010826330, 010826319, 010826328, 010826320, 010826338, 010826345, 010826327, 010826334 • 3♂♂; Malaysia, Sarawak (Borneo), 4^th^ Div. Gn. Mulu, RGS Exp.; ii–iii.1978; N.M. Collins; NHMUK010826343, 010826321, 010826352 • 1♀; Malaysia, Sarawak (Borneo), 1^st^ Div. Lubok, Jita, 1°12′N, 110°48′E; 6–10.xi.1976; P.S. Cranston; NHMUK010826370 • 1♂; Indonesia, Seram, Solea village, Malaise trap, forest; VIII.1987; coll. M.C. Day, BMNH(E) 1987-262; NHMUK010826350.

##### Distribution.

Brunei, Malaysia (Sarawak), Indonesia (Seram).

##### Etymology.

The specific name *carinatus*, meaning “keeled” or “carinate”, refers to the presence of the medio-longitudinal carina on posterior mesoscutum.

##### Remarks.

This species is similar to *W.nigratus*, but differs in the following: vein cu-a of hind wing curved (sinuate in *W.nigratus*); scutellum without apical spine in lateral view (with an apical spine in *W.nigratus*); and mesoscutum strigose apically (smooth in *W.nigratus*).

#### ﻿ Wilkinsonellus paracorpustriacolor

Taxon classificationAnimaliaHymenopteraBraconidae

Liu & Polaszek sp. nov.

D149A048-D2B3-50DB-9189-8A1670A4FA9B

https://zoobank.org/D39419AB-8B70-4133-9C02-21B25B0FD5CF

Fig. 4

##### Diagnosis.

**Figure 4.**
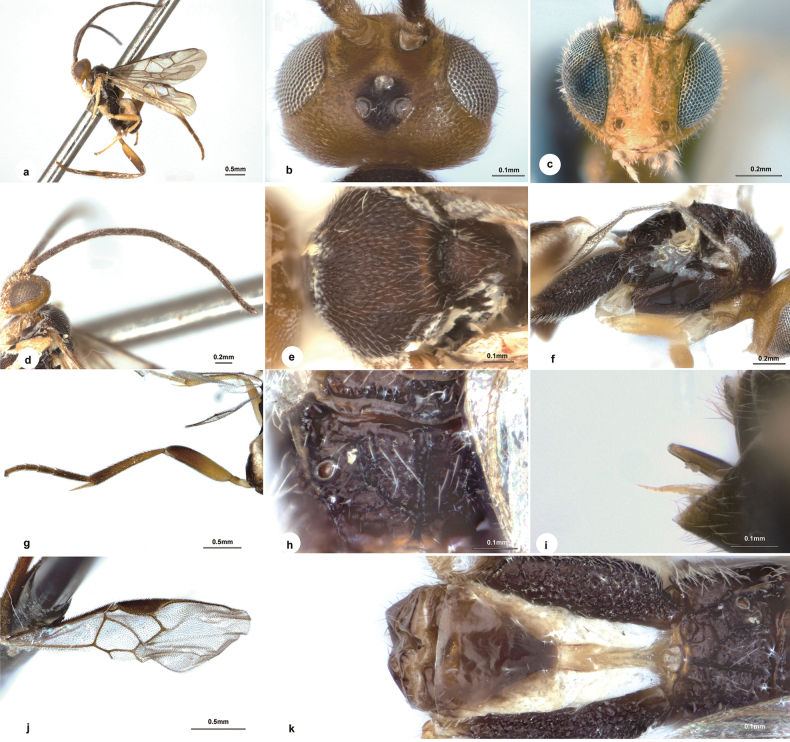
*Wilkinsonellusparacorpustriacolor* Liu & Polaszek, sp. nov., female **a** habitus, lateral view **b** head, dorsal view **c** head, frontal view **d** antenna **e** mesosoma, dorsal view **f** mesosoma, lateral view **g** hind leg **h** propodeum, dorsal view **i** ovipositor sheath **j** fore wing **k** metasoma, dorsal view.

Body 2.2 mm long, dark brown, except head yellow, petiole of T1 light yellow, T2–T7 brown and lateral part of T1 white (Fig. 4a); OOL:OD = 2.4 (Fig. 4b); face with weakly defined punctures, their intervals uneven (Fig. 4c); antenna with 1^st^ and 2^nd^ flagellomeres 2.8× and 3.0× longer than wide (Fig. 4d); mesoscutum with small punctures, intervals uneven with minute punctuation and larger than puncture’s diameter (Fig. 4e); scutellum with a spine apically in lateral view (Fig. 4f); propodeum with spiracles not enclosed by costulae anteriorly and posteriorly (Fig. 4h); mesopleuron largely smooth except indistinctly punctate on anterior and upper edges (Fig. 4f); vein r 0.7× 2-SR (Fig. 4j); metacoxa nearly surpassing T3, areolate-rugose on outer dorsal edge, weaker ventrally (Fig. 4f); petiole of T1 smooth, weakly constricted at anterior half; T2 subtriangular, 1.4× as wide as length in the middle; T3 1.5× as long as T2 (Fig. 4k); hypopygium a little broad (Fig. 4i).

##### Description.

**Female.** Body length 2.2 mm, fore wing length 2.4 mm (Fig. 4a).

***Head.*** 1.6× as wide as long, slightly wider than mesoscutum. Eyes 1.7× as long as temple in dorsal view. Temple slightly shiny with small punctures, rounded behind eyes in dorsal view. Vertex with weakly rugulose sculptures punctures, especially behind ocelli. Ocelli large, distance between fore and a posterior ocellus 0.8× as long as minor axis of a posterior ocellus, POL:OD:OOL = 1.3:1.0:2.4. Frons slightly depressed, nearly polished (Fig. 4b). Face slightly shiny, bulging medio-longitudinally, 0.9× as wide as high, with weakly defined punctures, their intervals irregular. Clypeus 2.8× wider than medial length, nearly smooth with superficial punctures. Length of malar space 1.3× width of mandible (Fig. 4c). Antenna with 1^st^ and 2^nd^ flagellomeres 2.8× and 3.0× longer than wide, eight apical flagellomeres missing (Fig. 4d).

***Mesosoma.*** Length:width:height = 1.7:1.2:1.0. Mesoscutum dull with small punctures, notauli indistinct, intervals irregular with minute punctation, narrowly polished along posterior margin. Scutellar sulcus wide and straight, with internal carinae (Fig. 4e). Scutellum dull, with rugulose punctures anteriorly, slightly polished posteriorly, slightly protruding above level of propodeum with a spine apically in lateral view (Fig. 4e, f). Propodeum 2.3× wider than high, dull with strong percurrent medio-longitudinal carina and adjacent oblique carinae which have obsolete carinae laterally; spiracles large, not enclosed by costulae (Fig. 4h). Mesopleuron shiny, largely smooth except indistinctly punctate on anterior and upper edges, sternaulus weakly crenulate, and with an oblique furrow attached at a right-angle to its anterior end (Fig. 4f).

***Wings.*** Fore wing: pterostigma narrow, 3.5× as long as its widest part; vein 1-R1 1.3× length of pterostigma; vein r arising from apical third of pterostigma, slightly longer than maximum width of pterostigma, 0.7× 2-SR; vein m-cu 2.9× as long as 2-SR+M, nearly half of 2-SR; vein 1-CU1 0.7× 2-CU1 and 1.8× cu-a (Fig. 4j). Hind wing: vannal lobe of normal size, posterior margin with short setae medially, basally and apically with longer setae, cu-a slightly curved.

***Legs.*** Metacoxa nearly surpassing T3, areolate-rugose on outer dorsal edge, with weaker sculpture ventrally (Fig. 4f). Metafemur 4.0× as long as its widest part. Inner spur of metatibia half length of metabasitarsus. Metabasitarsus 0.7× as long as combined length of tarsomeres 2–5 (Fig. 4g).

***Metasoma.*** 0.9× length of mesosoma. Petiole of T1 smooth, weakly constricted at anterior half, 2.3× longer than basal width, 5.0× longer than medial width, petiole with a groove reaching to anterior part of apical swollen area. T2 subtriangular, smooth, 1.4× as wide as length in the middle. T3 1.5× as long as T2, T3 and posterior tergites smooth and softer (Fig. 4k). Hypopygium slightly broadened, hardly exceeding apex of metasoma. Ovipositor sheath slightly protruding beyond metasoma, almost glabrous (Fig. 4i).

***Colour.*** Body dark brown, except head yellow, petiole of T1 light yellow, T2–T7 brown and lateral part of T1 white (Fig. 4a). Vertex with narrow striae present above occiput. Palpi white. Tibia spurs pale yellow. Antenna dark brown except scape and pedicel yellow. Fore and middle legs off-white to light yellow-brown, hind leg black to dark brown except trochanter pale yellow, basal half of femur and basal ¾ of tibia yellow-brown. Wing membranes hyaline, slightly infumate, fore wing with pterostigma brown, veins pale brown to brown.

**Male.** Similar to female except T1 longer with petiole at least 6.0× longer than medial width and ultimate flagellomere abruptly narrowed and pointed. Body length 2.2–2.6 mm.

##### Host.

Unknown.

##### Material examined.

(NHMUK) ***Holotype*** • 1♀; Malaysia, Sarawak (Borneo), Gn. Mulu; i–iv.1978; J.D. Holloway; NHMUK010639725. ***Paratypes*** • 1♀11♂; Malaysia, Sarawak (Borneo), 4^th^ div. Gn. Mulu; RGS Exp., N.M. Collins, Malaise trap; iii–vi.1978; NHMUK010826357, 010826358, 010826359, 010826360, 010826361, 010826362, 010826363, 010826364, 010826365, 010826366, 010826367, 010826368.

##### Distribution.

Malaysia (Sarawak).

##### Etymology.

The specific name *paracorpustriacolor* is in reference to the very similar appearance of the new species to *W. corpustriacolor*.

##### Remarks.

This species is very similar to *W.corpustriacolor*, but it differs in the following: vein m-cu of fore wing nearly half of 2-SR (about as long as 2-SR in *W.corpustriacolor*); mesoscutum with small punctures, intervals uneven with minute punctuation and larger than punture diameter (with dense areolate-rugose punctures in *W.corpustriacolor*); and propodeum without transverse carinae (with several transverse carinae attaching medio-longitudinal carina in *W.corpustriacolor*).

#### ﻿ Wilkinsonellus rugiscutum

Taxon classificationAnimaliaHymenopteraBraconidae

Liu & Polaszek sp. nov.

4AC0F081-955C-5747-ADC7-A9541C6D1670

https://zoobank.org/CE390E7C-9F5B-4986-94CC-7ABA566E9818

Fig. 5

##### Diagnosis.

**Figure 5.**
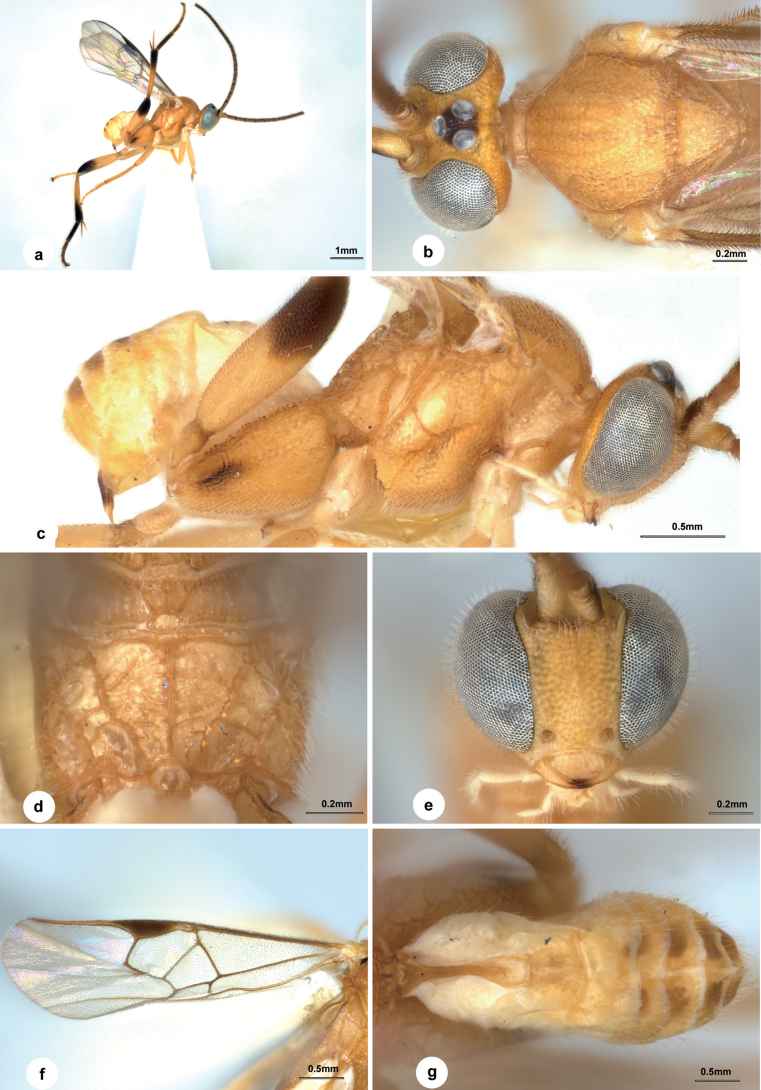
*Wilkinsonellusrugiscutum* Liu & Polaszek, sp. nov., female **a** habitus, lateral view **b** head and mesosoma, dorsal view **c** body, lateral view **d** propodeum, dorsal view **e** head, frontal view **f** fore wing **g** metasoma, dorsal view.

Body 3.7 mm long, bright yellow (Fig. 5a); OOL:OD = 0.4 (Fig. 5b); face wider dorsally (Fig. 5e); antenna with 1^st^ and 2^nd^ flagellomeres equal in length and 2.6× longer than wide (Fig. 5a); mesoscutum with notauli; scutellar sulcus with six carinae inside (Fig. 5b); scutellum with rugulose punctures, distinctly above level of propodeum with a spine apically in lateral view; propodeum 2.0× wider than high, spiracles enclosed by costulae anteriorly and posteriorly (Fig. 5d); mesopleuron with dense rugulose punctures except narrowly polished above sternaulus (Fig. 5c); vein r 1.4× 2-SR (Fig. 5f); metacoxa areolate-rugose on outer dorsal edge, with weaker sculpture laterally and ventrally (Fig. 5c); petiole of T1 strongly constricted medially, 12.5× longer than medial width; T2 trapezoid, smooth with shallow longitudinal grooves delimiting a median field that is indistinctly wider posteriorly; T3 0.9× as long as T2 (Fig. 5g); ovipositor sheath directed downwards (Fig. 5c).

##### Description.

**Female.** Body length 3.7 mm, fore wing length 3.8 mm (Fig. 5a).

***Head.*** 2.0× as wide as long, 1.2× slightly wider than mesoscutum. Eyes 4.6× longer than temple in dorsal view. Temple dull, with dense punctures, strongly constricted behind eyes in dorsal view. Vertex with shallow punctures excluding foveate areas along outside edge of posterior ocelli, a shallow depression present behind ocelli. Ocelli large, distance between fore and a posterior ocellus 0.4× as long as minor axis of a posterior ocellus, POL:OD:OOL = 1.0:2.3:1.0. Frons slightly depressed, nearly polished (Fig. 5b). Face little shiny, indistinctly bulging medio-longitudinally, 0.8× as wide as high, with dense punctures, their intervals uneven. Clypeus 2.3× wider than medial length, nearly smooth. Length of malar space as long as width of mandible (Fig. 5e). Antenna with 1^st^ and 2^nd^ flagellomeres equal in length and 2.6× longer than wide, three apical flagellomeres missing (Fig. 5a).

***Mesosoma.*** Length:width:height = 1.8:1.2:1.0. Mesoscutum dull with dense punctures, notauli indicated by slightly rugose punctures, intervals narrow, ridge-like. Scutellar sulcus wide and straight with six carinae inside (Fig. 5b). Scutellum dull, with rugulose punctures, distinctly above level of propodeum with a spine apically in lateral view (Fig. 5b, c). Propodeum 2.0× wider than high, a little shiny with strong percurrent medio-longitudinal carina and adjacent oblique carinae with thinner carinae at sides; spiracles large, enclosed by costulae anteriorly and posteriorly (Fig. 5d). Mesopleuron not shiny, with dense rugulose punctures except narrowly polished above sternaulus, the latter weakly crenulated and with an oblique furrow attaching right-angled to its anterior end (Fig. 5c).

***Wings.*** Fore wing: pterostigma narrow, 3.6× as long as its widest part; vein 1-R1 1.2× length of pterostigma; vein r arising from apical third of pterostigma, 1.4× longer than maximum width of pterostigma, 1.4× 2-SR; vein m-cu 0.7× as long as 2-SR+M, about half of 2-SR; vein 1-CU1 0.3× 2-CU1 and as long as cu-a (Fig. 5f). Hind wing: vannal lobe of normal size, posterior margin with short setae medially, basal and apical with longer setae, cu-a nearly straight.

***Legs.*** Metacoxa just surpassing T3, areolate-rugose on outer dorsal edge, with weaker sculpture laterally and ventrally (Fig. 5c). Metafemur 3.5× as long as its widest part. Inner spur of metatibia ^4^/_5_ length of metabasitarsus. Metabasitarsus 0.8× as long as combined length of tarsomeres 2–5 (Fig. 5a, c).

***Metasoma.*** Nearly as long as length of mesosoma. Petiole of T1 smooth, strongly constricted medially, 2.7× longer than basal width, 12.5× longer than medial width, petiole with a groove reaching to anterior half of apical swollen area. T2 trapezoidal, smooth, with shallow, longitudinal grooves delimiting a median field that is indistinctly widened posteriorly, 1.3× as wide as length in the middle. T3 0.9× as long as T2, T3, and posterior tergites smooth and softer (Fig. 5g). Hypopygium somewhat broad, hardly exceeding apex of metasoma. Ovipositor sheath slightly protruding beyond metasoma, pointing downwards, almost glabrous (Fig. 5c).

***Colour.*** Body bright yellow (Fig. 5a). Palpi white. Tibia spurs pale yellow. Antenna yellow-brown to dark brown. Fore and middle legs light yellow, hind leg yellow except black on macula of apical coxa, apical ^2^/_5_ of metafemur, apical ^1^/_3_ of metatibia and dark brown to yellow-brown on metatarsus. Wing membranes hyaline, indistinctly infumate, fore wing with pterostigma brown, veins pale brown to brown.

**Male.** Unknown

##### Host.

Unknown.

##### Material examined.

(NHMUK) ***Holotype*** • 1♀; Malaysia, Selangor, Serdang Carambola Farm; xi.1979; Gauld, Khashiyah; NHMUK010826369.

##### Distribution.

Malaysia (Selangor).

##### Etymology.

The specific name *rugiscutum* derives from the Latin “rugosus” and “scutum”, referring to the rugose punctate mesoscutum.

##### Remarks.

This species is similar to *W.longicentrus*, but differs in the following: fore wing with vein 1-CU1 shorter, 0.3× 2-CU1 (1-CU1 longer, over half length of 2-CU1 in *W.longicentrus*); POL shorter, less than half length of OD (POL longer, nearly as long as OD in *W.longicentrus*); and petiole of T1 longer, 12.5× longer than medial width (petiole shorter, at most 5× longer than medial width in *W.longicentrus*). It also resembles *W.amplus* from the Oriental region being pale in colour, but it can be easily differentiated from the latter by the black maculae on the apical metacoxa, and hind leg with less rugosity on lateral-dorsal part of metacoxa.

## ﻿Discussion

*Wilkinsonellus* is a distinctive genus of Microgastrinae, with 23 described species prior to this study. By adding three new species here, the Australo-Oriental region now has 84% (21) of the known species of this genus. *Wilkinsonellus* is pantropical, and the Australo-Oriental region appears to be a biodiversity hotspot for the genus, compared with the Afrotropical or Neotropical regions. However, there are still many undescribed species in collections, based on Fernández-Triana et al.’s (2020) observation. The phylogenetic position of the genus within Microgastrinae is still unclear, although it has been suggested as having a close relationship with *Diolcogaster* Ashmead based on morphology (Austin and Dangerfield 1992; Arias-Penna et al. 2014). More molecular data and analyses are needed to resolve its relationships.

## Supplementary Material

XML Treatment for Wilkinsonellus daira

XML Treatment for Wilkinsonellus amplus

XML Treatment for Wilkinsonellus carinatus

XML Treatment for Wilkinsonellus paracorpustriacolor

XML Treatment for Wilkinsonellus rugiscutum

## ﻿Additional information

### Author ORCIDs

Zhen Liu https://orcid.org/0000-0002-8670-0205

Andrew Polaszek https://orcid.org/0000-0002-7171-3353

### Data availability

All of the data that support the findings of this study are available in the main text.
